# Low prevalence of Epstein–Barr virus in incident gastric adenocarcinomas from the United Kingdom

**DOI:** 10.1038/sj.bjc.6600107

**Published:** 2002-03-04

**Authors:** D E Burgess, C B Woodman, K J Flavell, D C Rowlands, J Crocker, K Scott, J P Biddulph, L S Young, P G Murray

**Affiliations:** Department of Pathology, Division of Cancer Studies, The Medical School, University of Birmingham, Edgbaston, Birmingham B15 2TT, UK; CRC Institute for Cancer Studies, University of Birmingham, Edgbaston, Birmingham B15 2TT, UK; Centre for Cancer Epidemiology, University of Manchester, Kinnaird Road, Withington, Manchester M20 4QL, UK; Department of Histopathology, Birmingham Heartlands Hospital, Bordesley Green East, Birmingham B9 5SS, UK; Department of Histopathology, New Cross Hospital, Wolverhampton WV10 0QP, UK; Department of Epidemiology and Public Health, UCL (University College London), 1–19 Torrington Place, London WC1E 6BT, UK

**Keywords:** Epstein–Barr virus, gastric adenocarcinoma, EBERs

## Abstract

Epstein–Barr virus has been associated with a proportion of typical gastric adenocarcinomas. Here we report that the prevalence of Epstein–Barr virus in gastric adenocarcinomas from the United Kingdom is one of the lowest in the World. Gastric adenocarcinoma is another tumour whose association with Epstein–Barr virus varies with the population studied.

*British Journal of Cancer* (2002) **86**, 702–704. DOI: 10.1038/sj/bjc/6600107 www.bjcancer.com

© 2002 Cancer Research UK

## 

An early report that the Epstein–Barr virus (EBV) was present in the majority of lymphoepithelial-like carcinomas (LELCs) of the stomach, a rare form of gastric neoplasia (Shibata *et al*, 1991), was followed by the detection of EBV in 16% of typical gastric adenocarcinomas (Shibata and Weiss, 1992). The subsequent finding of monoclonal EBV episomes (Imai *et al*, 1994; Ott *et al*, 1994) and transforming proteins (zur Hausen *et al*, 2000) not only suggested an aetiological role for this virus in these tumours, but also the possibility that immunotherapy, for example CTL-based anti-EBV therapies (Rooney *et al*, 1998) might prolong survival for patients with virus-positive tumours. Before investing in such a strategy it is necessary to determine the numbers of patients who are likely to benefit. Here we report the largest survey of the prevalence of EBV in gastric adenocarcinomas undertaken outside Asia.

## MATERIALS AND METHODS

The base population comprised 497 consecutive patients who were first diagnosed with gastric adenocarcinoma between 1993–1999 and who had their tumours resected in one of three hospitals from the West Midlands, UK. This series was supplemented by an additional 69 patients with unresected disease in whom the diagnosis was made on gastric biopsy. Eight cases were excluded when histopathological review could not confirm the original diagnosis and 24 because there was insufficient material for the preparation of tissue arrays. EBV status was determined on the remaining 534 patients. All exclusions were from resected cases.

A 4 mm-diameter needle was used to sample representative tumour areas from paraffin blocks using a modification of the method described by Kononen *et al* (1998). Twenty cylindrical tumour cores were positioned in 5 mm holes cut from a 2.5×3 cm piece of paraffin-embedded liver tissue. Four micron thick sections cut from the array were adhered to Vectabond® coated slides and *in situ* hybridisation for the detection of the EBV-encoded RNAs (EBERs) performed according to standard methods (Wu *et al*, 1990). Positive controls which included cores from known EBV-positive gastric cancers were seeded into arrays. Seventy-five which tested negative for EBERs in tissue arrays, were re-evaluated by *in situ* hybridisation of the originating tissue blocks. U6 and sense control probes were included in all runs and assays were performed in duplicate.

## RESULTS

The mean age of patients in this series was 65.4 years (range 34–87 years). The male to female ratio was 2.2 : 1. Among 465 patients with resected disease 214 (46%) were classified as intestinal type according to the criteria of Lauren (1965), 112 (24%) were diffuse, 108 (23%) mixed and 31 (7%) were unclassifiable. One hundred and sixty-two (35%) of resected tumours involved the cardia, 289 (62%) the corpus/antrum, two (0.4%) the gastric stump and in 12 (2.6%) subsite was unknown. EBERs were detected in 9 out of 534 (1.7%) tumours in eight patients with resected disease and in one of those diagnosed on gastric biopsy. Both gastric stump cancers were EBV-positive.

## DISCUSSION

The prevalence of EBV-positive cancers in this series of gastric adenocarcinomas is substantially less than that reported elsewhere (Table 1

**Table 1:**
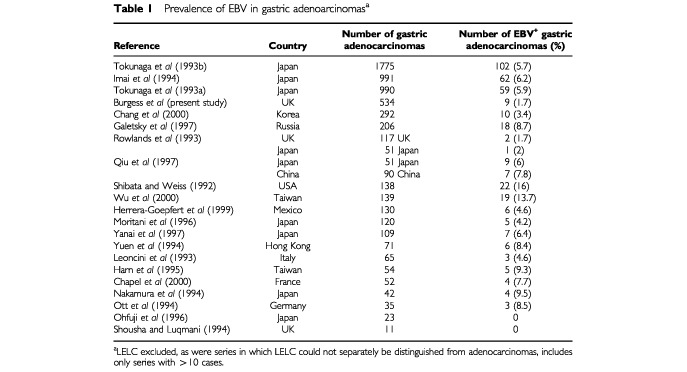
Prevalence of EBV in gastric adenoarcinomas^a^

). We believe this is not the result of sampling error in the preparation of the tissue arrays because re-evaluation of EBER-negative tumours using the originating tissue blocks revealed no false-negatives and our findings are similar to those reported in two smaller studies also undertaken in the UK which used tissue blocks when testing for the presence of EBERs (Rowlands *et al*, 1993; Shousha and Luqmani, 1994).

Although a higher detection rate of EBV-positive tumours has been reported in other European series, estimates are based on comparatively small numbers of cancers with only 10 tumours in total testing positive for EBV. No patient in our series was found to have a LELC. These tumours are strongly associated with EBV and both gastric LELC and LELCs at other sites are more common in Asia (Gaffey and Weiss, 1990). The higher rate of detection of EBV in gastric adenocarcinomas observed in large Asiatic series suggests that gastric tumours are another site of cancer where the strength of the association with EBV varies with the population studied.
